# Prognostic accuracy of time to sputum culture conversion in predicting cure in extensively drug-resistant tuberculosis patients: a multicentre retrospective observational study

**DOI:** 10.1186/s12879-022-07202-y

**Published:** 2022-03-02

**Authors:** Muhammad Abubakar, Nafees Ahmad, Muhammad Atif, Izaz Ahmad, Abdul Wahid, Asad Khan, Fahad Saleem, Abdul Ghafoor

**Affiliations:** 1 Department of Pharmacy Practice, Faculty of Pharmacy and Health Sciences, University of Balochistan, Quetta, Pakistan; 2 Department of Pharmacy Practice, Faculty of Pharmacy, The Islamia University of Bahawalpur, Bahawalpur, Pakistan; 3 Department of Biology, Syed Babar Ali School of Science and Engineering, Lahore University of Management Sciences, Lahore, Pakistan; 4 National TB Control Program, Islamabad, Pakistan

**Keywords:** Cure, High dose isoniazid, Sensitivity, Specificity, Sputum culture conversion, XDR-TB

## Abstract

**Background:**

There was a lack of information about prognostic accuracy of time to sputum culture conversion (SCC) in forecasting cure among extensively drug-resistant tuberculosis (XDR-TB) patients. Therefore, this study evaluated the prognostic accuracy of SCC at various time points in forecasting cure among XDR-TB patients.

**Methods:**

This retrospective observational study included 355 eligible pulmonary XDR-TB patients treated at 27 centers in Pakistan between 01-05-2010 and 30-06-2017. The baseline and follow-up information of patients from treatment initiation until the end of treatment were retrieved from electronic nominal recording and reporting system. Time to SCC was analyzed by Kaplan–Meier method, and differences between groups were compared through log-rank test. Predictors of time to SCC and cure were respectively evaluated by multivariate Cox proportional hazards and binary logistic regression analyses. A p-value < 0.05 was considered statistically significant.

**Results:**

A total of 226 (63.6%) and 146 (41.1%) patients respectively achieved SCC and cure. Median time to SCC was significantly shorter in patients who achieved cure, 3 months (95% confidence interval [CI]: 2.47–3.53), than those who did not (median: 10 months, 95% CI: 5.24–14.76) (p-value < 0.001, Log-rank test). Patient’s age > 40 years (hazards ratio [HR] = 0.632, p-value = 0.004), baseline sputum grading of scanty, + 1 (HR = 0.511, p-value = 0.002), + 2, + 3 (HR = 0.523, p-value = 0.001) and use of high dose isoniazid (HR = 0.463, p-value = 0.004) were significantly associated with early SCC. Only SCC at 6 month of treatment had statistically significant association with cure (odds ratio = 15.603, p-value < 0.001). In predicting cure, the sensitivities of SCC at 2, 4 and 6 months were respectively 41.8% (95%CI: 33.7–50.2), 69.9% (95%CI: 61.7–77.2) and 84.9% (95%CI: 78.1–90.3), specificities were respectively, 82.8% (95%CI: 76.9–87.6), 74.6% (95%CI: 68.2–80.4) and 69.4% (95%CI: 62.6–75.5) and prognostic accuracies were respectively 65.9% (95%CI: 60.7–70.8), 72.7% (95%CI: 67.7–77.2) and 75.8% (95%CI: 71.0–80.1).

**Conclusion:**

In forecasting cure, SCC at month 6 of treatment performed better than SCC at 2 and 4 months. However, it would be too long for clinicians to wait for 6 months to decide about the regimen efficacy. Therefore, with somewhat comparable prognostic accuracy to that SCC at 6 month, using SCC at 4 month of treatment as a prognostic marker in predicting cure among XDR-TB patients can decrease the clinicians waiting time to decide about the regimen efficacy.

**Supplementary Information:**

The online version contains supplementary material available at 10.1186/s12879-022-07202-y.

## Background

Extensively drug-resistant tuberculosis (XDR-TB) was previously defined as “TB caused by *Mycobacterium tuberculosis* (MTB) concurrently resistant to *rifampicin (RIF)*, *isoniazid (INH)*, any *fluoroquinolones (FQs)* and at least one of the three second-line injectable (SLIs) drugs i.e. *amikacin (AM), kanamycin (KM)* and *capreomycin (CM*)” [1]. However, its definition is now modified to “TB caused by MTB concurrently resistant to *RIF, INH,* any *FQs* and either *bedaquiline (BDQ)* or *linezolid (LZD)*, or both” [1]. As XDR-TB is resistant to the most effective first and second line anti-TB drugs (SLD), it is the most difficult to treat form of TB [1–10]. Patients with XDR-TB are treated for prolonged times with multiple first and SLD of lower or unproven efficacy. This subsequently results in relatively lower global treatment success rate (43%) in XDR-TB patients (2017 cohort) than patients with MDR (58%, 2017 cohort) and drug susceptible TB (85%, 2018 cohort). In the published literature, the reported treatment success rate of various individual cohorts of XDR-TB patients ranged from 4 to 65% [2–10].

Despite recent advances in TB diagnostic tests in the form of automated molecular test, treatment response in both drug susceptible and drug resistant TB (DR-TB) is still assessed using microbiological techniques such as sputum smear examination and culture [11]. A new bacteriological response term *bacteriological conversion* defined as “a situation in a patient with bacteriologically confirmed TB where at least two consecutive cultures for DR-TB and drug susceptible-TB or smears for drug susceptible-TB, taken on different occasions at least 7 days apart, are negative” has been recently introduced for assessing the effectiveness of anti-TB treatment [12]. However, as in routine management of DR-TB, sputum smear and culture are done on monthly basis [13], therefore, sputum culture conversion (SCC) defined as “two successive negative culture specimen obtained at the space of at least one month following a baseline positive culture” [14, 15] plays a cardinal role in observing the treatment response, predicting the effectiveness of the regimen, identifying the constraints and deciding about the treatment duration and treatment outcomes of DR-TB patients [14, 16–18]. In addition to clinical settings, SCC remains the most commonly used surrogate marker for evaluating the efficacy of anti-TB drugs in clinical trials [16]. There are multiples studies which have evaluated the validity of time to SCC in forecasting treatment outcomes among multidrug resistant TB (MDR-TB) patients [14, 16–19]. Some studies have reported that SCC at 2 months has low sensitivity in predicting cure. Many of MDR-TB patients who did not achieve SCC at 2 months of treatment had successful end treatment outcomes [14, 16]. Javaid et al., and Kurbatova et al., have reported that, SCC at 6 month of treatment among MDR-TB patients had an overall stronger association with treatment success than SCC at 2 month [14, 16]. Similarly, another study conducted among MDR-TB patients in China has concluded that SCC at 6 month of treatment was a comparatively more accurate prognostic marker of predicting treatment success than SCC and 2 and 3 month of treatment [17]. Likewise, in a study conducted at Hunan Chest Hospital, China and Gondar University Hospital, Ethiopia, Alene et al., have reported that the optimum SCC time points to predict treatment success among MDR-TB patients were SCC between 4 and 6 months of treatment [11]. On the other hand, some studies have reported SCC at 2 months of treatment as predictor of cure among MDR-TB patients [18, 19]. Although limited information is available on this topic among MDR-TB patients, however there was complete lack of this information among XDR-TB patients. Therefore, the current study was conducted with the objectives to evaluate predictors of time to SCC and cure, prognostic accuracy of SCC at various time points in predicting cure among XDR-TB patients.

## Methods

### Study population, settings and design

Pakistan is DR-TB 5th high burden country in the world. Programmatic management of DR-TB (PMDT) in Pakistan was started way back in 2010, and at present there are 33 functional PMDT units in the country [10, 20]. In the current study, we retrospectively evaluated the record of all those culture confirmed pulmonary XDR-TB patients who were enrolled for treatment at 27 PMDT units in the country between 01-05-2010 and 30-06-2017 (Additional file 1: Table S1). XDR-TB patients with extra-pulmonary disease, negative sputum culture at baseline visit, unknown drug-resistance pattern, and with end-treatment outcome of lost to follow-up (LTFU) were excluded from the study.

The following operational definitions were adopted in the current study. XDR-TB patients were those “who were infected by MTB concurrently resistant to *RIF, INH* any *FQs* and at least one of the three SLIs i.e. *AM, KM* and *CM*” [10]. SCC was defined as “two successive negative cultures obtained at the space of at least one month following a baseline positive culture”. Time to SCC was defined as “the time in days from the initiation of XDR-TB treatment to the date of sample collection of the first of two successive negative cultures” [14]. The WHO guidelines’ recommended and National TB Control Program of Pakistan (NTP) adopted criterion was used for defining TB treatment outcomes [21]. Sensitivity of SCC in predicting “cure” was defined as “the proportion of patients with SCC by month 2, 4 and 6 among those who were declared cured” [14]. Specificity of SCC in predicting “cure” was defined as “the proportion of patients without SCC by month 2, 4 and 6 among those who did not achieve cure i.e. died or were declared treatment failures”. Prognostic accuracy of SCC at various time points of treatment in predicting end treatment outcome was calculated as “correctly predicted cured cases plus correctly predicted death and treatment failure cases divided by the total case number.”[14].

### Diagnosis and treatment of XDR-TB patients

The protocols adopted for drug susceptibility testing (DST) and treatment of XDR-TB patients at the current study sites have been published elsewhere [10]. In line with NTP guidelines [21], all suspected pulmonary DR-TB patients referred to the treatment centres were respectively assessed for MTB, resistance to *RIF* and *INH* by examining two sputum samples through direct sputum smear microscopy, Xpert *MTB/RIF* (Cepheid, Sunnyvale, CA, United States) and line probe assay (LPA). After confirmation of *RIF* resistant-TB, patients were put on empirical treatment regimen recommended by NTP guidelines [21]. Meanwhile, for phenotypic DST, their sputum samples were sent to the reference laboratories. At these laboratories, DST against anti-TB drugs was carried out using Agar proportion method on enriched Middlebrook 7H10 medium (BBL; Beckton Dickinson, Sparks, MD, United States) at the following concentrations: *RIF* (1 µg/ml), *INH* (0.2 µg/ml), *streptomycin* (2 µg/ml), *ethambutol (EMB)* (5 µg/ml), *AM* (4 µg/ml), *KM* (5 µg/ml), *CM* (4 µg/ml), ethionamide *(ETO)* (5 µg/ml), *ofloxacin (OFX)* (2 µg/ml) and *LFX* (1 µg/ml). Whereas, DST for *pyrazinamide (PZA)* at a concentration of 100 µg/ml was done by using BACTEC Mycobacterial Growth Indicator Tube (MGIT, BD, Sparks, MD, USA) [10, 13, 22–24]. Upon availability of DST results, patients diagnosed with XDR-TB were shifted to individualized treatment regimens (ITRs). In compliance with NTP guidelines, ITRs were principally consisted of an *SLI*, in priority the one to which the MTB was sensitive, a high generation *FQ*, all available likely effective Group-4s-line anti-TB drugs (SLD) [*ETO, cycloserine (CS) and para-amino salicylic acid (PAS*)], *EMB* if the strain was susceptible, *PZA* and ≥ 2 of the Group-5 drugs [*BDQ, delamanid (DLM), LZD, clofazimine (CFZ), amoxicillin/clavulanate, imipenem/cilastatin* + *clavulanate, meropenem* + *clavulanate, high-dose INH, clarithromycin (CLR), thioacetazone*). Patients were treated for at least 20 months with a minimum of 18 months post SCC. All patients received SLIs for a minimum of 8–12 months. Patients were treated as outpatients. Their treatment adherence was observed by trained treatment supporters, assessed by clinicians on monthly visits and guaranteed by a home DOTS (directly observed treatment, short-course) linkage facilitator by home visits [10, 21].

### Data collection and statistical analysis

Each PMDT unit in the country shares its monthly data with NTP Islamabad through Electronic Nominal Recording and Reporting System (ENRS). ENRS is a combined excel sheet of the following four main TB recoding and reporting registers (i) basic management unit TB register, (ii) second-line TB treatment register (iii) laboratory register for smear microscopy and Xpert MTB/Rif and (iv) laboratory register for culture, Xpert MTB/RIF and DST. ENRS contains information about the patients’ sociodemographic characteristics like age, gender, marital status, residence and smoking, history of TB treatment, treatment centre, duration, regimen and outcome of previous episode of TB treatment, presence of any concurrent medical condition, history of any SLD used, results of Xpert MTB/Rif and LPA, phenotypic DST results, monthly weight, sputum smear microscopy and culture results, treatment regimen for DR-TB and treatment outcomes [10, 13, 25]. We retrieved the abovementioned data from ENRS through a purpose developed data collection form.

Statistical Package for Social Sciences (version 26) was used for analyzing data. Time to SCC was analysed using the Kaplan–Meier method, and differences between groups were assessed using the log-rank test [11, 16, 18]. Bivariate and multivariate Cox proportional hazards regression analyses were used to identify predictors of time to SCC [14, 26, 27]. Those cases who did not achieve SCC were censored one month before their last sputum culture date. Sensitivity, specificity and prognostic accuracy of SCC at 2, 4 and 6 month in predicting end treatment outcomes were also assessed. In order to envision the effects of SCC at different time points on the balance between sensitivity and specificity, Receiver Operating Characteristic Curves (ROC) were plotted. Multivariate binary logistic regression analysis (MVBLRA) was conducted to evaluate the variables which had statistically significant association with cure. After checking for correlation, all those variables which had an association with cure at the p-value of < 0.2 were included in MVBLRA. Statistical significance was taken at a p-value < 0.05.

## Results

**Fig. 1:**
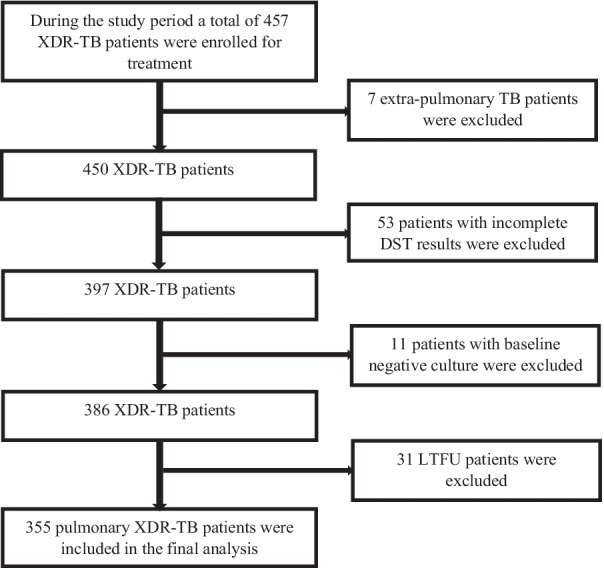
Flow chart of study participants included in the study. *DST* drug susceptibility testing, *LTFU* lost to follow-up, *XDR-TB* extensively drug resistant tuberculosis

During the study period i.e. from 01-05-2010 to 30-06-2017, a total of 457 patients were enrolled for XDR-TB treatment at 27 treatment centers in the country. Among them, 355 XDR-TB patients fulfilled the inclusion criteria and were included in final analysis (Fig. 1).

### Socio-demographic and clinical characteristics of study participants

Mean age of the patients was 32.99 ± 14.54 years and about half of them were males (52.7%). Majority patients had a history of previous TB treatment (92.3%), no history of SLD use (62.3%), were previously not treated for MDR-TB (64.8%) and did not suffer from co-morbidity (84.2%).

### Drug resistance pattern and therapeutic regimen

Patients were resistant to a median of 7 drugs (range 6–9). Resistance was highest for *AM* (75.8%), followed by *KM* (75.2%), *EMB* (74.1%), *PZA* (70.4%), *CM* (61.7%) and *ETO* (12.7%). A total of 44.8% were resistant to all SLIs and 40.3% were resistance to all five first-line anti-TB drugs.

Patients were treated with a median of 10 drugs (range: 9–11). The most commonly used drug was *PZA* (99.4%), followed by *CS* (97.7%), *ETO* (97.5%), *PAS* (88.7%),*CM* (73.5), *LZD* (68.7%), *moxifloxacin (MFX)* (66.8%), *co-amoxiclav* (66.5%), *CLR* (58.9%), *CFZ* (53.8%), *LFX* (32.1%), *AM* (20%), *EMB* (18.3%), high dose *INH* (9.9%), *BDQ* (9.3%), *DLM* (2.3%) and *KM* (1.7%).

### Time to sputum culture conversion, cure and their predictors

**Fig. 2:**
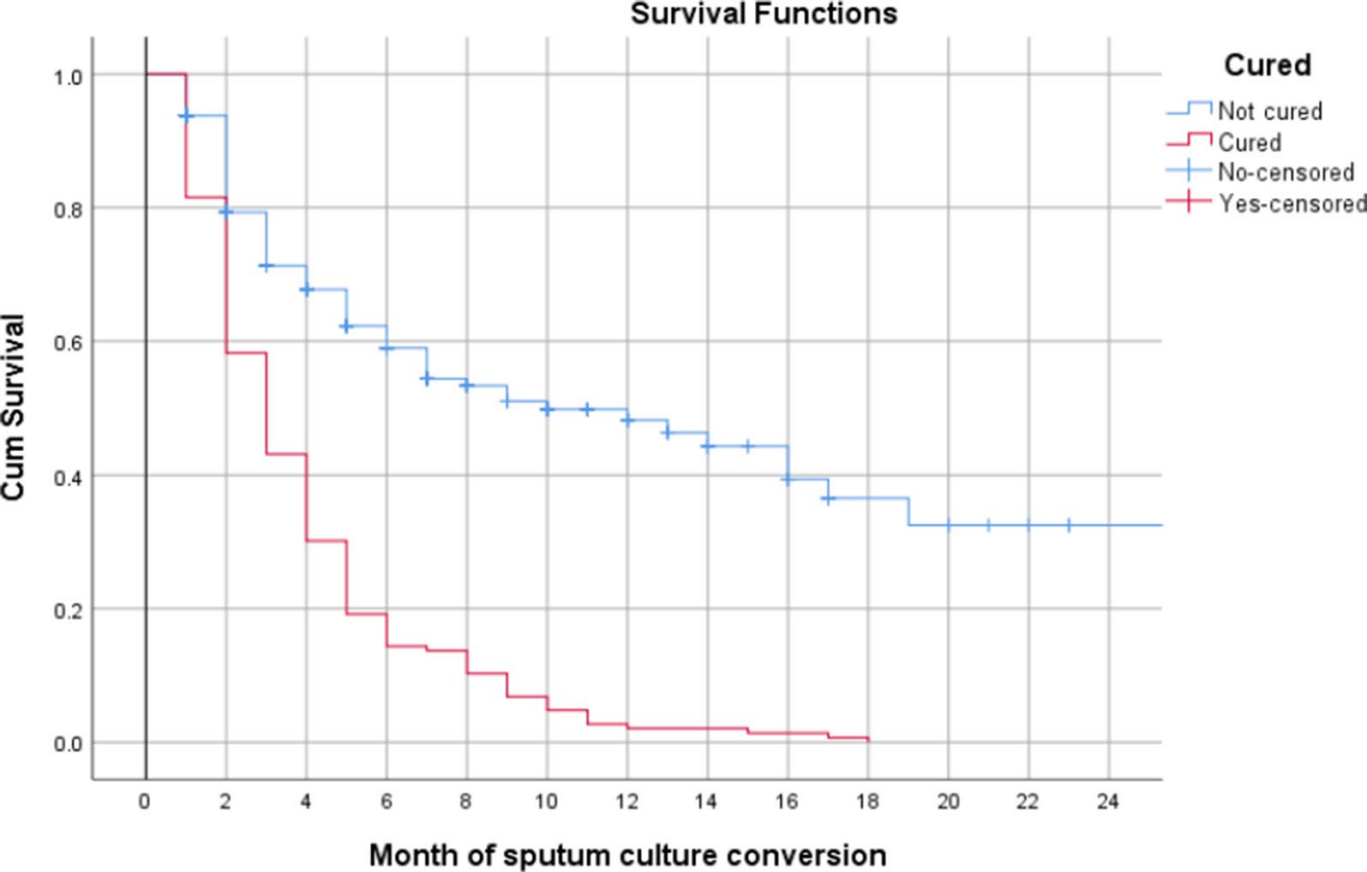
Time to sputum culture conversion among XDR-TB patients by treatment outcomes (cured vs not cured), N = 355

A total of 226 (63.6%) patients achieved SCC. The mean time to SCC was 91 days (Interquartile range: 59–156). Ninety seven patients (27.3%), were culture negative at second month of treatment, 155 (43.6%) at 4^th^ and 188 (53.0%) at 6^th^ month. Among patients who achieved cure, the median time to SCC was significantly shorter, 3 months (95% CI 2.47–3.53), compared with patients who were not cured (median: 10 months, 95% CI 5.24–14.76) (p-value < 0.001, Log-rank test) (Fig. 2).

The results of Multivariate Cox Proportional Hazards Model revealed that Patient’s age > 40 years (Hazards ratio [HR] = 0.632, p-value = 0.004), baseline sputum grading of scanty, + 1 (HR = 0.511, p-value = 0.002), + 2, + 3 (HR = 0.523, p-value = 0.001) and use of high dose isoniazid (HR = 0.463, p-value = 0.004) were significantly associated with early SCC (Table 1).

**Table 1:** Predictors of time to sputum culture conversion

Variable	SCC No. (%)	Univariate analysis HR (95%CI)	p-value	Multivariate analysis HR (95%CI)	p-value
Gender					
Female Male	107 (63.7) 119 (63.6)	Referent 0.900 (0.693–1.170)	0.433		
Age (years)					
≤ 40 > 40	173 (67.3) 53 (54.1)	Referent 0.632 (0.464–0.862)	0.004	Referent 0.597 (0.414–0.859)	0.006
Baseline body weight (kg)					
< 40 ≥ 40	72 (58.1) 154 (66.7)	Referent 0.993 (0.751–1.315)	0.963		
Marital status					
Single Married Widow	78 (68.4) 143 (61.4) 5 (2.2)	Referent 0.827(0.627–1.090) 0.478 (0.193–1.183)	0.178 0.110	Referent 0.964 (0.707–1.314) 0.752 (0.281–2.013)	0.815 0.571
History of TB treatment					
No Yes	20 (83.3) 206 (62.2)	Referent 0.677 (0.428–1.073)	0.097	Referent 0.886 (0.541–1.450)	0.629
History of MDR-TB treatment					
No Yes	153 (66.5) 73 (58.4)	Referent 0.684 (0.517–0.905)	0.008	Referent 0.805 (0.599–1.081)	0.150
Baseline sputum smear grading					
Negative Scanty* + 1† + 2‡& + 3§ Not available	38 (67.9) 68 (61.8) 117 (63.6) 3 (60)	Referent 0.652 (0.438–0.971) 0.630 (0.437–0.910) 0.393 (0.121–1.276)	0.035 0.014 0.120	Referent 0.511 (0.336–0.777) 0.523 (0.353–0.777) 0.443 (0.132–1.479)	0.002 0.001 0.186
Co-morbidity					
No Yes	191 (63.9) 35 (62.5)	Referent 0.850 (0.592–1.222)	0.380		
Number of resistant drugs					
4–6 7–8 > 8	71 (71) 95 (57.2) 60 (67.4)	Referent 0.612 (0.449–0.836) 0.749 (0.530–1.060)	0.002 0.103	Referent 0.689 (0.457–1.039) 1.012 (0.572–1.790)	0.075 0.967
Resistance to all five FLD					
No Yes	132 (62.3) 94 (65.7)	Referent 0.828 (0.634–1.082)	0.168	Referent 0.862 (0.546–1.361)	0.525
Resistance to ethambutol					
No Yes	60 (65.2) 166 (63.1)	Referent 0.757 (0.562–1.019)	0.066	Referent 0.920 (0.622–1.359)	0.675
Resistance to pyrazinamide					
No Yes	72 (68.6) 154 (61.6)	Referent 0.810 (0.611–1.073)	0.142	Referent 1.106 (0.752–1.628)	0.608
Resistance to streptomycin					
No Yes	88 (62.9) 138 (64.2)	Referent 0.888 (0.667–1.136)	0.387		
Resistance to ethionamide					
No Yes	200 (64.5) 26 (57.8)	Referent 0.670 (0.444–1.012)	0.057	Referent 0.632 (0.399–1.003)	0.051
Use of moxifloxacin					
No Yes	68 (57.6) 158 (66.7)	Referent 1.453 (1.092–1.934)	0.010	Referent 1.449 (0.991–2.118)	0.056
Use of para-amino salicylic acid					
No Yes	19 (47.5) 207 (65.7)	Referent 1.430 (0.892–2.292)	0.137	Referent 1.028 (0.619–1.707)	0.914
Use of clarithromycin					
No Yes	90 (61.6) 136 (65.1)	Referent 0.919 (0.703–1.200)	0.535		
Use of co-amoxiclav					
No Yes	71 (59.7) 155 (65.7)	Referent 1.086 (0.819–1.438)	0.567		
Use of bedaquiline					
No Yes	208 (64.6) 18 (54.5)	Referent 1.208 (0.746–1.958)	0.442		
Use of delamanid					
No Yes	222 (64.0) 4 (50.0)	Referent 0.734 (0.273–1.975)	0.540		
Use of clofazimine					
No Yes	109 (66.5) 117 (61.3)	Referent 1.227 (0.943–1.596)	0.128	Referent 0.884 (0.607–1.288)	0.522
Use of linezolid					
No Yes	67 (60.4) 159 (65.2)	Referent 1.288 (0.992–1.635)	0.159	Referent 0.991 (0.703–1.396)	0.958
Use of amikacin					
No Yes	181(63.7) 45 (63.4)	Referent 1.045 (0.754–1.449)	0.791		
Use of capreomycin					
No Yes	58 (61.7) 168 (64.4)	Referent 0.995 (0.738–1.342)	0.796		
Use of high dose isoniazid					
No Yes	210 (65.6) 16 (45.7)	Referent 0.648 (0.389–1.077)	0.094	Referent 0.463 (0.267–0.802)	0.004
Use of ethambutol					
No Yes	186 (64.1) 40 (61.5)	Referent 1.243 (0.883–1.751)	0.213		

^*^Scanty = 1–9 AFB (Acid fast bacilli)/100 HPF (High power field); † + 1 = 10–99 AFB/100 HPF); ‡ + 2 = 1–9 AFB/HPF; § + 3 > 9 AFB/HPF

CI = Confidence interval; FLD = First line anti-TB drugs, HR: Hazards ratio, MDR: Multidrug resistant, SLD = Second line anti-TB drugs, SCC = Sputum culture conversion

In the current study, a total of 146 (41.1%) patients achieved cure. In MVBLRA, SCC at month 6 of treatment (OR: 15.603, 95%CI: 6.168–39.467) emerged as the only predictor of cure (Table 2).

**Table 2:** Predictors of cure

Variable	Cured No. (%)	Univariate analysis OR (95%CI)	p-value	Multivariate analysis OR (95%CI)	p-value
Gender					
Female Male	73 (43.5) 73 (39)	Referent 0.833 (0.546–1.273)	0.399		
Age (years)					
≤ 40 > 40	112 (43.6) 34 (34.7)	Referent 0.688 (0.424–1.115)	0.129	Referent 0.990 (0.488–2.009)	0.979
Baseline body weight (kg)					
< 40 ≥ 40	42 (33.9) 104 (45)	Referent 1.599 (1.016–2.516)	0.042	Referent 1.661 (0.922–2.994)	0.091
Marital status					
Single Married Widow	54 (47.4) 90 (38.6) 2 (25)	Referent 0.699 (0.445–1.099) 0.370 (0.072–1.913)	0.121 0.236	Referent 0.737 (0.394–1.378) 0.396 (0.045–3.484)	0.339 0.404
History of TB treatment					
No Yes	14 (58.3) 132 (39.9)	Referent 0.474 (0.204–1.098)	0.082	Referent 0.527 (0.186–1.492)	0.228
History of MDR-TB treatment					
No Yes	97 (42.2) 49 (39.2)	Referent 0.884 (0.567–1.379)	0.587		
Baseline sputum smear grading					
Negative Scanty* + 1† + 2‡ + 3§ Not available	27 (48.2) 51 (46.4) 67 (75.7) 1 (20)	Referent 0.928 (0.487–1.768) 0.615 (0.336–1.125) 0.269 (0.028–2.558)	0.821 0.115 0.253	Referent 1.144 (0.491–2.665) 0.603 (0.276–1.318) 0.709 (0.061–8.238)	0.756 0.205 0.783
Co-morbidity					
No Yes	120 (40.1) 26 (46.4)	Referent 1.293 (0.728–2.295)	0.380		
Number of resistant drugs					
4–6 7–8 > 8	46 (46) 54 (32.5) 46 (51.7)	Referent 0.566 (0.340–0.943) 1.256 (0.709–2.226)	0.029 0.435	Referent 0.523 (0.257–1.066) 1.010 (0.365–2.791)	0.075 0.985
Resistance to all five FLD					
No Yes	79 (37.3) 67 (46.9)	Referent 1.484 (0.965–2.283)	0.072	Referent 1.798 (0.760–2.674)	0.058
Resistance to ethambutol					
No Yes	39 (42.4) 107 (40.7)	Referent 0.932 (0.576–1.508)	0.775		
Resistance to pyrazinamide					
No Yes	43 (41) 103 (41.2)	Referent 1.010 (0.536–1.606)	0.965		
Resistance to streptomycin					
No Yes	53 (37.9) 93 (43.3)	Referent 1.251 (0.810–1.934)	0.313		
Resistance to ethionamide					
No Yes	127 (41) 19 (42.2)	Referent 1.053 (0.559–1.984)	0.873		
Resistance to SLIs					
No Yes	85 (43.4) 61 (38.4)	Referent 0.813 (0.531–1.245)	0.341		
Use of moxifloxacin					
No Yes	46 (39.0) 100 (42.2)	Referent 1.142 (0.728–1.793)	0.563		
Use of para-amino salicylic acid					
No Yes	11(27.5) 135 (42.9)	Referent 1.977 (0.954–4.099)	0.067	Referent 1.539 (0.620–3.823)	0.330
Use of clarithromycin					
No Yes	64 (43.8) 82 (39.2)	Referent 0.827 (0.539–1.270)	0.386		
Use of co-amoxiclav					
No Yes	47 (39.5) 99 (41.9)	Referent 1.107 (0.706–1.735)	0.657		
Use of bedaquiline					
No Yes	135 (41.9) 11 (33.3)	Referent 0.693 (0.325–1.476)	0.342		
Use of clofazimine					
No Yes	71 (43.3) 75 (39.3)	Referent 0.847 (0.554–1.294)	0.442		
Use of linezolid					
No Yes	43 (45.7) 103 (42.2)	Referent 1.155 (0.730–1.827)	0.538		
Use of amikacin					
No Yes	117 (41.2) 29 (40.8)	Referent 0.986 (0.581–1.673)	0.957		
Use of capreomycin					
No Yes	38 ( 40.4) 108 (41.4)	Referent 1.040 (0.644–1.681)	0.872		
Use of high dose isoniazid					
No Yes	138 (43.1) 8 (22.9)	Referent 0.391 (0.172–0.887)	0.025	Referent 0.433 (0.57–1.195)	0.106
Use of ethambutol					
No Yes	118 (40.7) 28 (43.1)	Referent 1.103 (0.640–1.900)	0.724		
SCC at month 2					
No Yes	85 (32.9) 61 (62.9)	Referent 3.449 (2.119–5.612)	< 0.001	Referent 0.658 (0.310–1.394)	0.274
SCC at month 4					
No Yes	44 (22) 102 (65.8)	Referent 6.823 (4.259–10.931)	< 0.001	Referent 1.037 (0.388–2.773)	0.942
SCC at month 6					
No Yes	21 (12.7) 125 (66.1)	Referent 12.770 (7.348–21.923)	< 0.001	Referent 15.603 (6.168–39.467)	< 0.001

^*^Scanty = 1–9 AFB (Acid fast bacilli)/100 HPF (High power field); † + 1 = 10–99 AFB/100 HPF); ‡ + 2 = 1–9 AFB/HPF; § + 3 > 9 AFB/HPF, CI = Confidence interval; FLD = First line anti-TB drugs; OR = Odds ratio, SCC = Sputum culture conversion; SLD = Second line anti-TB drugs

In predicting cure, the sensitivities of SCC at 2, 4 and 6 months of treatment were respectively 41.8% (95% CI: 33.7–50.2), 69.9% (95% CI: 61.7–77.2) and 84.9% (95% CI: 78.1–90.3), whereas, specificities were respectively, 82.8% (95% CI: 76.9–87.6), 74.6% (95% CI: 68.2–80.4) and 69.4% (95% CI: 62.6–75.5). Whereas, the overall prognostic accuracy of SCC in predicting end treatment outcome at 2, 4 and 6 months of treatment were respectively 65.9% (95% CI: 60.7–70.8), 72.7% (95% CI: 67.7–77.2) and 75.8% (95% CI: 71.0–80.1) (Table 3).

**Table 3:** Association of sputum culture conversion at different time points with cure

Month of treatment	Cured No. (%)		Odds ratio (95% Cl)	p-value*	Sensitivity (95% Cl)	Specificity (95% Cl)	Accuracy (95% Cl)
	No	Yes					
2-month							
Did no convert Converted	173 (67.1) 36 (37.1)	85 (32.9) 61 (62.9)	Referent 3.4 (2.1–5.6)	< 0.001	41.8 (33.7–50.2)	82.8 (76.9–87.6)	65.9 (60.7–70.8)
4-month							
Did no convert Converted	156 (78.0) 53 (34.2)	44 (22.0) 102 (65.8)	Referent 6.8 (4.2–10.9)	< 0.001	69.9 (61.7–77.2)	74.6 (68.2–80.4)	72.7 (67.7–77.2)
6-month							
Did no convert Converted	145 (86.8) 64 (34.0)	22 (13.2) 124 (66.0)	Referent 12.7 (7.4–21.9)	< 0.001	84.9 (78.1–90.3)	69.4 (62.6–75.5)	75.8 (71.0–80.1)

*CI* confidence interval,

*Univariate binary logistic regression analysis

**Fig. 3:**
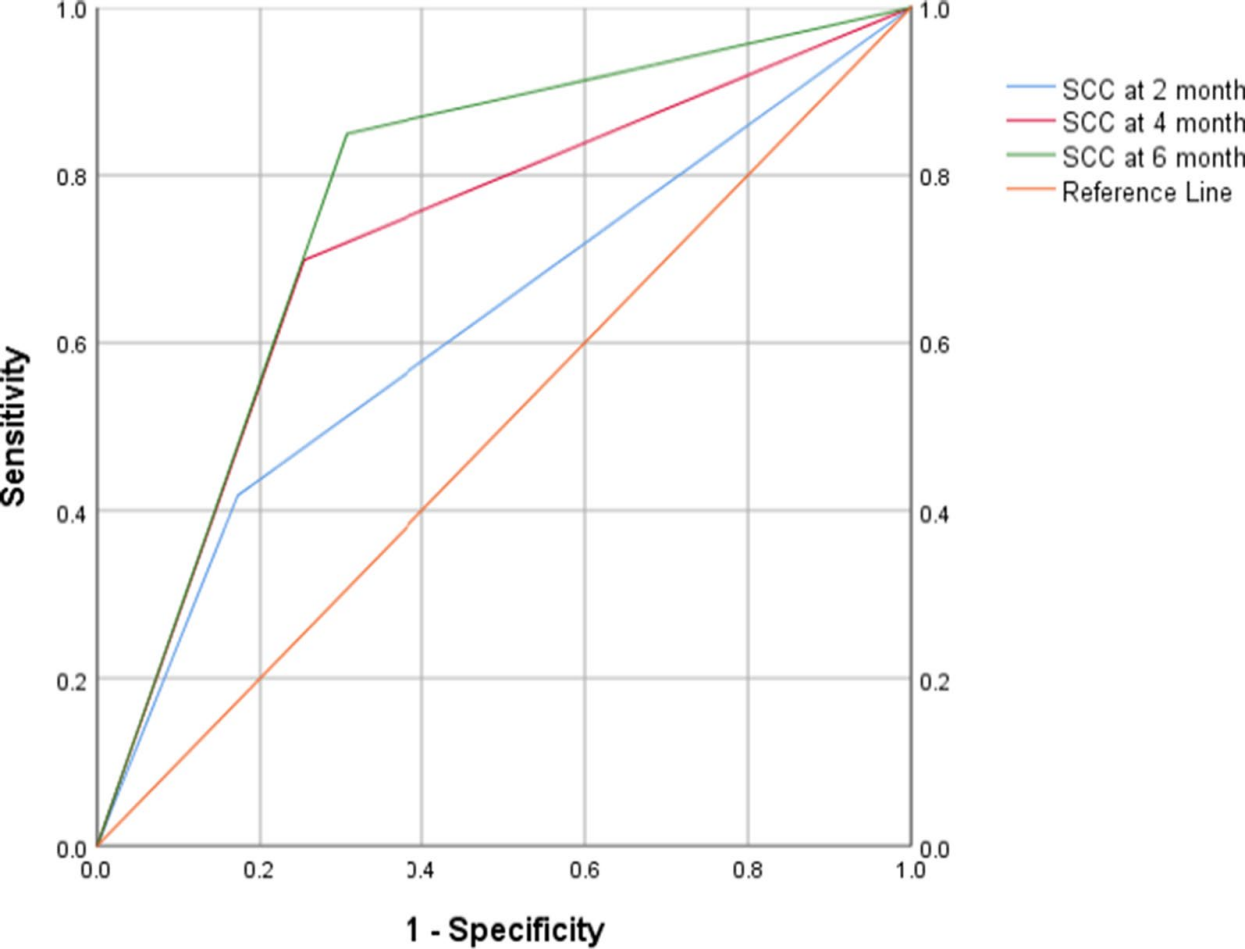
Receiver operating characteristic curve for prognostic performance of time to sputum culture conversion in predicting cure

ROC visualizes the effect of using different time points of SCC on the balance between sensitivity and specificity in predicting cure. ROC analysis by non-parametric method revealed a comparatively better discrimination power of the SCC on month 6 (Area under cure [AUC] = 0.772, 95% CI: 0.721–0.822, p-value < 0.001) than month 4 (AUC = 0.723, 95% CI: 0.668–0.778, p-value < 0.001) and month 2 (AUC = 0.623, 95% CI: 0.562–0.683, p-value < 0.001) (Fig. 3).

## Discussion

This study included a total of 355 culture confirmed pulmonary XDR-TB patients treated at 27 PMDT units in Pakistan. In addition to evaluating the factors associated with achieving early SCC and cure, to the best of our knowledge, this is the first study which analyzed the prognostic accuracy of SCC on different time points in predicting end treatment outcomes in XDR-TB patients. In the current cohort, a total 63.6% patients achieved SCC and 41.1% were cured. The median time to SCC were 91 days. Ninety seven patients (27.3%) were culture negative at second month of treatment, 155 (43.6%) at 4th and 188 (53.0%) at 6th month. The proportion of culture negative patients within first 6 months of treatment (53%) was higher than that reported from India (43.7%) [5], but lower than the range (83–89%) reported elsewhere [28–30].

In the present study, patients who were > 40 years old, had baseline positive sputum smear status of scanty, 1 +, 2 + and 3 + and received high dose *INH* were significantly less likely to achieve early SCC than their counterparts. The finding of older age as a risk factor for delayed culture conversion is in line with previous studies which have reported older age as a risk factor of delayed culture conversion and poor treatment outcomes among MDR/XDR-TB patients [8, 10, 24, 26, 31, 32]. This could be due to the combination of multiple risk factors like compromised immunity, concurrent comorbidities, complex medication schedule and poor compliance with the regimen [8, 10, 24, 26, 31, 32]. Although in the previous published studies no significant association has been reported between the uses of high dose *INH* and delayed SCC in DR-TB patients, but it has been associated with death among XDR-TB patients [10]*.* Based on the assumption that high dose *INH* could be effective against MTB strains with low-level *INH* resistance because of mutations in the inhA promotor at positions 8, 15 or 16 [33], WHO guidelines suggested the use of high dose INH in the treatment of MDR/XDR-TB patients [34]. However, there is a general consent that treatment with high-dose *INH* cannot overcome the high level *INH* resistance resulting from mutation in the katG gene at position 315 [33, 35]. A study conducted in Republic of Moldova which included 2638 MTB strains found that mutation in the katG gene at position 315 was present in > 88% of the examined strains [35]. Nevertheless, the current finding of negative association between the use of high dose *INH* and early SCC among XDR-TB patients should be interpreted with the limitation that only 9.9% patients were treated with the regimens containing high dose *INH*. Furthermore, due to retrospective nature of the data collection, we were unable to find out the reason of using high dose *INH* in only one tenth of the study participants, but we suspect the severity of disease as the major cause of receiving high dose *INH* which could have resulted in delayed SCC in these patients. However, it is suggested that in the absence of comprehensive molecular drug resistance testing, the indiscriminate use of high dose *INH* in the treatment of DR-TB should be discouraged [10, 35]. The significantly less likelihood of achieving early culture conversion in patients with baseline positive sputum smear (scanty, 1 +, 2 + and 3 +) is in line with the studies in which baseline positive and high smear grading emerged as predictors of delayed culture conversion in MDR/XDR-TB patients [27, 29, 36, 37]. The delay in SCC in patients with baseline positive smear and high smear grading could be due to high bacillary load suggesting a stronger infectivity, advanced lung lesions, and well spread disease which make these patients less responsive to optimal regimen and requires longer time to clear the bacillary load [27, 29, 36].

In the present study, while predicting cure, the sensitivities of SCC at 2, 4 and 6 months of treatment were respectively 41.8%, 69.9% and 84.9% and specificities were respectively 82.8%, 74.6% and 69.4%, whereas, the overall prognostic accuracies were respectively 65.9%, 72.7% and 75.8%. On the basis of these findings, if SCC at 2 month is used as a surrogate marker for predicting cure in XDR-TB patients, it would accurately predict end treatment outcomes in only 65.9% patients, and a total of 58.2% patients would be misjudged as patients with unsuccessful outcomes (death and treatment failure). This carries the risk of underestimating the effectiveness of the regimen, compromising its efficacy by replacing the effective drugs, its early termination, and unnecessary drug therapy [14, 16]. Whereas, the highest sensitivity and accuracy observed for SCC at 6 month of treatment (84.9% and 75.8%, respectively) suggest that using it as a surrogate marker would accurately predict end treatment outcomes in 75.8% patients and reduce the proportion of cured patients misjudged as treatment failures to 15.1%. This signifies that a regimen which is unable to produce SCC at 6 months of treatment has a very little chance of producing cure in XDR-TB patients. This finding was in line with studies in which SCC at 6 month had high sensitivity in predicting cure in multidrug resistant TB (MDR-TB) patients [14, 16, 17] and was also supported by the emergence of SCC at 6 month as the only predictor of cure in multivariate analysis in the current cohort. Those patients who were culture negative by 6 month of treatment, were 15.6 times more likely to achieve cure than their counterparts (Table 3). Similar stronger association between culture negativity at month 6 of treatment and successful outcomes among MDR-TB patients have been reported elsewhere [16, 17]. However, comparatively low specificity of SCC at 6 months of treatment (69.4%) is a matter of concern and would overrate the effectiveness of the regimen [16]. If SCC at 6 month of treatment is taken as a prognostic marker, 30.6% patients with eventual outcome of death and treatment failure would be misjudged as achieving cure. On the other hand, the highest specificity observed for SCC at 2 month of treatment (82.2%) advocates that, if SCC at this time point is taken as a proxy marker for predicting cure, only 17.8% of patients who did not achieve SCC by 2 month of treatment would eventually achieve cure. These findings suggest that although SCC at 2 month of treatment gives some assurance about the effectiveness of regimen, but due to its low sensitivity, lack of SCC at this time point may be premature to declare the regimen ineffective and modify or terminate it, unless the patient’s clinical condition is deteriorating [14, 16]. On the other hand, it would be too long for clinicians to wait for SCC at 6 months and not reassessing and modifying the regimen, again, depending upon the patient’s clinical condition [14, 16]. In current study, the combined sensitivity (69.6%), specificity (74.6%) and accuracy (72.7%) of SCC at 4 month of treatment were somewhat comparable to those of SCC at 6 month of treatment (84.9%, 69.9%, 75.8%, respectively). Therefore, using SCC at 4 month as a prognostic marker for predicting cure in XDR-TB patients would accurately predict end treatment outcome in 72.7% patients, could decrease the clinicians’ waiting time to decide about the effectiveness of XDR-TB treatment regimen and do timely modifications if needed [14].

## Conclusions

The current findings demonstrate that in predicting cure among XDR-TB patients, SCC at 6 months of treatment performed better than SCC at 2 and 4 months. However, its relatively low specificity (69.4%) and long waiting period for clinicians to decide about the effectiveness of the regimen are matters of concern. With somewhat comparable prognostic accuracy to that of SCC at 6 month and shorter waiting period to decide about the effectiveness of the regimen, it would be rational to use SCC at 4 month of treatment as prognostic marker in predicting cure among XDR-TB patients. However, if the patient clinical condition is not deteriorating, clinicians may wait for achieving sputum culture negativity within 6 months to decide about the effectiveness of the regimen.

Large number of XDR-TB patients who were diagnosed, treated and reported under uniform protocols, and data collection from a standard ENRS are the major strengths of this study. However, retrospective observational design and lack of information about lung cavitation, adverse events and their impact on treatment outcomes are the potential limitations of this study. Furthermore, as large number patients (n = 51) with unknown drug resistance pattern, culture negative results on the baseline visit (n = 11) and who were LTFU (n = 31) were excluded from the study, this might have induced bias in findings of the current study.

## Supplementary Information

**Additional file 1: Table S1. **Study site wise distribution of study participants.

## Data Availability

All data gathered or analyzed during this study are included in the article. The raw data on which conclusions of this manuscript rely is available upon request. Please contact Nafees Ahmad at nafeesuob@gmail.com.

## Abbreviations

AM: Amikacin
AUC: Area under curve
BDQ: Bedaquiline
CI: Confidence interval
CM: Capreomycin
CLR: Clarithromycin
CFZ: Clofazimine
CS: Cycloserine
DLM: Delamanid
DR-TB: Drug-resistant tuberculosis
DST: Drug susceptibility testing
EMB: Ethambutol
ETO: Ethionamide
XDR-TB: Extensively DR-TB
FQ: Fluoroquinolones
ENRS: Electronic nominal recording and reporting system
HR: Hazards ratio
ITR: Individualized treatment regimen
INH: Isoniazid
KM: Kanamycin
LFX: Levofloxacin
LTFU: Loss to follow up
MFX: Moxifloxacin
MVBLRA: Multivariate binary logistic regression analysis
NTP: National TB Control Program
OFX: Ofloxacin
OR: Odds ratio
PAS: Para-amino salicylic acid
ROC: Receiver operating characteristic curve
RIF: Rifampicin
SCC: Sputum culture conversion
WHO: World Health Organization

## References

[1] World Health Organization. WHO consolidated guidelines on drug-resistant tuberculosis treatment. World Health Organization; 2019. License: CC BY-NC-SA 3.0 IGO. https://apps.who.int/iris/handle/10665/311389. 30946559

[2] Abbate E, Vescovo M, Natiello M (2012). Successful alternative treatment of extensively drug-resistant tuberculosis in Argentina with a combination of linezolid, moxifloxacin and thioridazine. J Antimicrob Chemother.

[3] Alene KA, Yi H, Viney K (2017). Treatment outcomes of patients with multidrug-resistant and extensively drug resistant tuberculosis in Hunan Province, China. BMC Infect Dis.

[4] He X-c, Tao N-n, Liu Y, Zhang X-x, Li H-c (2017). Epidemiological trends and outcomes of extensively drug-resistant tuberculosis in Shandong, China. BMC Infect Dis.

[5] Prajapati K, Mishra V, Desai M, Solanki R, Naik P (2017). Treatment outcome of patients having extensively drug-resistant tuberculosis in Gujarat, India. Int J Mycobacteriol.

[6] Gallo J, Pinhata J, Simonsen V, Galesi V, Ferrazoli L, Oliveira R (2018). Prevalence, associated factors, outcomes and transmission of extensively drug-resistant tuberculosis among multidrug-resistant tuberculosis patients in São Paulo, Brazil: a cross-sectional study. Clin Microbiol Infect.

[7] Yuengling KA, Padayatchi N, Wolf A (2018). Effect of antiretroviral therapy on treatment outcomes in a prospective study of extensively drug resistant tuberculosis (XDR-TB) HIV co-infection treatment in KwaZulu-Natal, South Africa. J Acquir Immun Defic Syndr..

[8] Frank M, Adamashvili N, Lomtadze N, et al., editors. Long-term follow-up reveals high posttreatment mortality rate among patients with extensively drug-resistant tuberculosis in the country of Georgia. Open Forum Infect Dis. 2019;6(4). 10.1093/ofid/ofz152. 10.1093/ofid/ofz152 PMC6483133 31041349

[9] Makhmudova M, Maxsumova Z, Rajabzoda A, Makhmadov A, Van Den Hof S, Mirtskhulava V (2019). Risk factors for unfavourable treatment outcomes among rifampicin-resistant tuberculosis patients in Tajikistan. Int J Tuberc Lung Dis.

[10] Abubakar M, Ahmad N, Ghafoor A (2021). Treatment outcomes of extensively drug-resistant tuberculosis in Pakistan: a countrywide retrospective record review. Front Pharmacol.

[11] Alene KA, Viney K, Yi H, McBryde ES, Yang K, Bai L (2018). Comparison of the validity of smear and culture conversion as a prognostic marker of treatment outcome in patients with multidrug-resistant tuberculosis. PLoS ONE.

[12] World Health Organization. Meeting report of the WHO expert consultation on drug-resistant tuberculosis treatment outcome definitions, 17–19 November 2020. World Health Organization. https://apps.who.int/iris/handle/10665/340284. License: CC BY-NC-SA 3.0 IGO. 2021.

[13] Naz F, Ahmad N, Wahid A, Ahmad I, Khan A, Abubakar M (2021). High rate of successful treatment outcomes among childhood rifampicin/multidrug-resistant tuberculosis in Pakistan: a multicentre retrospective observational analysis. BMC Infect Dis.

[14] Javaid A, Ahmad N, Afridi AK (2018). Validity of time to sputum culture conversion to predict cure in patients with multidrug-resistant tuberculosis: a retrospective single-center study. Am J Trop Med Hyg.

[15] Basit A, Ahmad N, Khan AH (2014). Predictors of two months culture conversion in multidrug-resistant tuberculosis: findings from a retrospective cohort study. PLoS ONE.

[16] Kurbatova EV, Cegielski JP, Lienhardt C (2015). Sputum culture conversion as a prognostic marker for end-of-treatment outcome in patients with multidrug-resistant tuberculosis: a secondary analysis of data from two observational cohort studies. Lancet Respir Med.

[17] Lu P, Liu Q, Martinez L (2017). Time to sputum culture conversion and treatment outcome of patients with multidrug-resistant tuberculosis: a prospective cohort study from urban China. Eur Respir J.

[18] Holtz TH, Sternberg M, Kammerer S (2006). Time to sputum culture conversion in multidrug-resistant tuberculosis: predictors and relationship to treatment outcome. Ann Intern Med.

[19] Ige O, Akindele Y, Oladokun R, Adebiyi O. Sputum culture conversion among the first cohorts of MDR-TB patients managed in Nigeria at a tertiary care hospital. Eur Respir J. 2014;44(Suppl 58).

[20] Wahid A, Ahmad N, Ghafoor A (2021). Effectiveness of shorter treatment regimen in multidrug-resistant tuberculosis patients in Pakistan: a multicenter retrospective record review. Am J Trop Med Hyg.

[21] National TB Control Program. National guidelines for the programmatic management of drug-resistant tuberculosis (PMDT) 2014. Islamabad, Pakistan: National TB Control Program.

[22] Javaid A, Ahmad N, Khan AH, Shaheen Z (2017). Applicability of the World Health Organization recommended new shorter regimen in a multidrug-resistant tuberculosis high burden country. Eur Respir J.

[23] Ahmad N, Javaid A, Sulaiman SAS, Ming LC, Ahmad I, Khan AH (2016). Resistance patterns, prevalence, and predictors of fluoroquinolones resistance in multidrug resistant tuberculosis patients. Braz J Infect Dis.

[24] Ahmad N, Javaid A, Basit A, Afridi A, Khan M, Ahmad I (2015). Management and treatment outcomes of MDR-TB: results from a setting with high rates of drug resistance. Int J Tuberc Lung Dis.

[25] Atif M, Ahmad W, Ahmad N, Malik I, Sarwar S (2020). Treatment outcomes among multidrug-resistant TB patients in Bahawal Victoria Hospital, Bahawalpur, Pakistan: a retrospective record review. Trans R Soc Trop Med Hyg.

[26] Li Q, Lu M, Hsieh E, Wu L, Wu Y, Wang M (2020). Time to sputum culture conversion and its predictors among patients with multidrug-resistant tuberculosis in Hangzhou, China: a retrospective cohort study. Medicine.

[27] Yihunie Akalu T, Muchie KF, Alemu GK (2018). Time to sputum culture conversion and its determinants among multi-drug resistant tuberculosis patients at public hospitals of the Amhara Regional State: a multicenter retrospective follow up study. PLoS ONE.

[28] Maretbayeva SM, Rakisheva AS, Adenov MM (2021). Culture conversion at six months in patients receiving bedaquiline-and delamanid-containing regimens for the treatment of multidrug-resistant tuberculosis. Int J Infect Dis.

[29] Franke MF, Khan P, Hewison C (2021). Culture conversion in patients treated with bedaquiline and/or delamanid a prospective multicountry study. Am J Respir Crit Care Med.

[30] Gao M, Gao J, Xie L (2021). Early outcome and safety of bedaquiline-containing regimens for treatment of MDR-and XDR-TB in China: a multicentre study. Clin Microbiol Infect.

[31] Khan I, Ahmad N, Khan S, Muhammad S, Khan SA, Ahmad I (2019). Evaluation of treatment outcomes and factors associated with unsuccessful outcomes in multidrug resistant tuberculosis patients in Baluchistan province of Pakistan. J Infect Public Health.

[32] Kurbatova EV, Taylor A, Gammino VM, Bayona J, Becerra M, Danilovitz M (2012). Predictors of poor outcomes among patients treated for multidrug-resistant tuberculosis at DOTS-plus projects. Tuberculosis.

[33] Domínguez J, Boettger E, Cirillo D, Cobelens F, Eisenach K, Gagneux S (2016). Clinical implications of molecular drug resistance testing for Mycobacterium tuberculosis: a TBNET/RESIST-TB consensus statement. Int J Tuberc Lung Dis.

[34] World Health Organization. WHO treatment guidelines for drug resistant tuberculosis. 2016; Geneva, Switzerland: World Health Organization. WHO/HTM/TB/2016.04. https://apps.who.int/iris/bitstream/handle/10665/250125/9789241549639-eng.pdf.

[35] Chesov D, Ciobanu N, Lange C, Heyckendorf J, Crudu V (2017). High-dose isoniazid in the shorter-course multidrug-resistant tuberculosis regimen in the Republic of Moldova. Eur Respir J.

[36] Liu Q, Lu P, Martinez L (2018). Factors affecting time to sputum culture conversion and treatment outcome of patients with multidrug-resistant tuberculosis in China. BMC Infect Dis.

[37] Russkikh A, Korotych O, Sereda Y, et al. Factors associated with culture conversion among adults treated for pulmonary extensively drug-resistant tuberculosis during 2018–2019 in the Russian Federation: an observational cohort study. Monaldi Arch Chest Dis. 2021;91(1). 10.4081/monaldi.2021.1678. 10.4081/monaldi.2021.1678 33470087

