# Supplementary material for: Prognostic accuracy of time to sputum culture conversion in predicting cure in extensively drug-resistant tuberculosis patients: a multicentre retrospective observational study
Source: BMC Infect Dis. 2022 Mar 2;22:204. doi: 10.1186/s12879-022-07202-y (PMC8889712; doi:10.1186/s12879-022-07202-y)
Supplement: Supplementary file 1 — Additional file 1: Table S1. Study site wise distribution of study participants. [file 12879_2022_7202_MOESM1_ESM.docx]

**Additional file 1**

**Table S1. Study site wise distribution of study participants**

| **S. No** | **PMDT site** | **Province** | **No. (%)** |
| --- | --- | --- | --- |
| 1 | Gulab Devi Hospital Lahore | Punjab | 32 (9.0) |
| 2 | Leprosy Hospital Rawalpindi | Punjab | 35 (9.9) |
| 3 | Nishter Hospital Multan | Punjab | 30 (8.5) |
| 4 | Jinnah Hospital Lahore | Punjab | 11 (3.1) |
| 5 | Govt. Samli Sanatorium Hospital Murree | Punjab | 16 (4.5) |
| 6 | Mayo Hospital Lahore | Punjab | 12 (3.4) |
| 7 | District Headquarter Hospital Sargodha | Punjab | 10 (2.8) |
| 8 | Sheikh Zayed Hospital Rahim Yar Khan | Punjab | 8 (2.3) |
| 9 | Bahawal Victoria Hospital Bahawalpur | Punjab | 7 (2.0) |
| 10 | District Headquarter Hospital Faisalabad | Punjab | 6 (1.7) |
| 11 | Allama Iqbal Memorial DHQ Hospital Sialkot | Punjab | 3 (0.8) |
| 12 | Military Hospital Rawalpindi | Punjab | 3 (0.8) |
| 13 | Abbas Institute of Medical Sciences Hospital Muzaffarabad | AJK | 6 (1.7) |
| 14 | Indus Hospital Karachi | Sindh | 42 (11.8) |
| 15 | Ojha Institute of Chest Disease Karachi | Sindh | 41 (11.5) |
| 16 | Institute of Chest Disease Kotri | Sindh | 13 (3.7) |
| 17 | Ghulam Muhammad Meher Civil Hospital Sukkur | Sindh | 13 (3.7) |
| 18 | District Headquarter Hospital Mirpurkhas | Sindh | 11 (3.1) |
| 19 | Jinnah Postgraduate Medical Center Karachi | Sindh | 2 (0.6) |
| 20 | District Headquarter Hospital Tharparkar | Sindh | 3 (0.8) |
| 21 | Chandka Medical College Larkana | Sindh | 1 (0.3) |
| 22 | Peoples Medical College Nawabshah | Sindh | 1 (0.3) |
| 23 | Fatima Jinnah Chest Hospital Quetta | Balochistan | 7 (2.0) |
| 24 | Lady Reading Hospital Peshawar | KPK | 26 (7.3) |
| 25 | Mufti Mehmood Memorial Teaching Hospital DI Khan | KPK | 8 (2.3) |
| 26 | Ayub Teaching Hospital Abbottabad | KPK | 6 (1.7) |
| 27 | Saidu Sharif Teaching Hospital Swat | KPK | 2 (0.6) |
| **Total** | | | **355 (100)** |

AJK=Azad Jammu and Kashmir, KPK= Khyber Pukhtoonkhwa, PMDT= Programmatic management of drug-resistant tuberculosis unit
